# Microdevice for plasma separation from whole human blood using bio-physical and geometrical effects

**DOI:** 10.1038/srep26749

**Published:** 2016-06-09

**Authors:** Siddhartha Tripathi, Y. V. BalaVarun Kumar, Amit Agrawal, Amit Prabhakar, Suhas S. Joshi

**Affiliations:** 1 Indian Institute of Technology Bombay, Powai, Mumbai 400076, India; 2 Indian Institute of Information Technology, Devghat, Jhalwa, Allahabad 211012, India

## Abstract

In this research work, we present a simple and efficient passive microfluidic device for plasma separation from pure blood. The microdevice has been fabricated using conventional photolithography technique on a single layer of polydimethylsiloxane, and has been extensively tested on whole blood and enhanced (upto 62%) hematocrit levels of human blood. The microdevice employs elevated dimensions of about 100 μm; such elevated dimensions ensure clog-free operation of the microdevice and is relatively easy to fabricate. We show that our microdevice achieves almost 100% separation efficiency on undiluted blood in the flow rate range of 0.3 to 0.5 ml/min. Detailed biological characterization of the plasma obtained from the microdevice is carried out by testing: proteins by ultra-violet spectrophotometric method, hCG (human chorionic gonadotropin) hormone, and conducting random blood glucose test. Additionally, flow cytometry study has also been carried on the separated plasma. These tests attest to the high quality of plasma recovered. The microdevice developed in this work is an outcome of extensive experimental research on understanding the flow behavior and separation phenomenon of blood in microchannels. The microdevice is compact, economical and effective, and is particularly suited in continuous flow operations.

Blood is one of the easiest gateways to assess internal performance of the human body. Blood can be broadly thought as being a complex composition of cells suspended in plasma. Red Blood Cells (RBCs), White blood Cells (WBCs) and platelets occupy a volume of about 45%^1^,^2^. The remaining volume is occupied by the liquid portion of blood (plasma), which is straw-yellow in color^1^,^2^. The hematocrit (Hct) or packed cell volume (PCV) or erythrocyte volume fraction is the proportion of blood volume that is occupied by red blood cells^2^. Separation of cells from plasma is one of the most basic, essential and vital step in the field of disease diagnostics^3^,^4^. The separation is carried out so as to minimize the interference of cells in the process of analyte detection^3^. Conventionally, plasma separation is carried out via centrifugation. Although the process of centrifugation is easy and widely used, the process is tedious, time consuming and involves multiple stages of blood handling^3^,^5^. Also, coupling of centrifugation with a lab-on-a-chip device is a non-trivial issue and invalidates the entire testing being completed at the micro-scale^3^,^4^,^5^,^6^,^7^. In this regard, microfluidic devices have the potential to address these issues in an effective manner. In this study, we present a microdevice capable of replacing the conventional plasma separation technique i.e. achieving separation of plasma at a micro-scale. A complete study on the systematic evolution and performance evaluation of the microdevice has been carried out. Therefore, this study is well aligned with the current worldwide efforts of developing microdevices that incorporate several laboratory functions on a single chip of a few square millimeters to a few square centimeters in size.

In general, the techniques of blood plasma separation in microfluidic devices can be classified as passive or active. Active methods rely on external forces for cell separation^8^,^9^,^10^,^11^,^12^,^13^. On the other hand, passive methods incorporate inertial, bio-physical and geometrical effects. The passive techniques which include sedimentation, filtration, lateral displacement and hydrodynamic flow separation, are preferred over active methods^3^,^5^,^14^,^15^,^16^,^17^. Among the passive techniques, the hydrodynamic flow separation techniques are relatively advantageous compared to the other passive methods^5^. Various hydrodynamic microdevices reported in literature utilize bio-physical effects such as Fahraeus effect^18^,^19^,^20^,^21^ and Zweifach-Fung bifurcation law^1^,^22^. It is observed that as blood flows in a tube of diameter less than 300 μm, the cells have a tendency to migrate towards the center of the tube resulting in the formation of a cell free layer along the tube walls^23^,^24^,^25^. Due to the formation of cell free layer, the tube hematocrit becomes lower than the feed hematocrit. This effect is known as ‘Fahraeus effect’. The ensuing hematocrit in the tube is a function of tube diameter and feed hematocrit. The tube hematocrit is known to decrease with a decrease in tube diameter^18^,^19^,^20^,^21^. Another relevant bio-physical effect is Zweifach-Fung bifurcation law, which governs the cell behavior at a bifurcation. As per this law, upon encountering a bifurcation, the cells tend to flow in the branch with the higher flow rate, provided that certain specific conditions are met^1^,^22^. Extensive studies have demonstrated that disproportionate distribution of RBCs indeed occurs at a bifurcation^26^,^27^,^28^,^29^,^30^.

In addition to the above mentioned bio-physical effects, certain geometrical factors such as constriction-expansion^31^ and bends^32^ have also been employed in the design of blood plasma separation microdevices. More details on the performance of devices based on hydrodynamic separation can be found elsewhere^5^. Despite having a reasonable understanding of the role of bio-physical and geometrical effects, researchers have been striving hard to integrate these aspects into a simple, effective and efficient microfluidic device which possesses the capability to separate plasma from whole blood. This quest for a simple and efficient microdevice has rapidly accelerated from the past decade because obtaining cell free plasma is a basic requirement in disease diagnostics. Most of the microdevices presented in literature are capable of achieving high separation efficiency of plasma extraction, but they require dilution of blood samples^31^,^32^,^33^,^34^,^35^,^36^,^37^,^38^,^39^,^40^. That is, only limited success has been reported with microdevices working on whole human blood. A major challenge in this endeavor stems from the fact that more than 5 million cells are to be separated per micro-liter of blood. Other major challenges faced in separating whole blood in microdevices are discussed elsewhere^5^,^33^. A T-filtration microdevice reported by Yang *et al.*^34^ provided a separation efficiency of 100% with sheep blood. Jaggi *et al.*^35^ experimented with high flow rate, high aspect ratio microdevice employing Hct 45% to obtain an efficiency of 37%. Virrallel *et al.*^36^ designed a hybrid microdevice and employed elevated temperatures to obtain efficiency of 97% with blood of 40% Hct. However elevating the fluid temperature without damaging the cells remains a challenge^41^. A hybrid microdevice by Kerhoas *et al.*^3^,^37^ showed 99% separation efficiency with whole blood. However, some factors which may limit the viability of these microdevices, especially at high hematocrit content, include low microchannel dimensions (clogging), very low rate of plasma extraction, and complexity in design and fabrication technique. Therefore, our survey indicates that there exists sufficient scope for betterment in the process of plasma separation from whole human blood.

Another issue with employing diluted blood samples in a plasma separation microdevice may be with respect to the quality of plasma extracted^38^,^39^,^40^. Understandably, there is uncertainty associated with analyte detection from these diluted blood plasma samples. Particular interest is to ensure presence of analytes/biomarkers from plasma obtained. Therefore, it becomes inevitable to biologically characterize plasma, especially the plasma obtained using diluted blood samples^4^,^37^.

In our previous studies on ‘simple T’^38^ and ‘Tau’ microchannels^39^,^40^, we presented several designs which provided excellent separation efficiency on moderate to high hematocrit content blood. But, with an increase in the hematocrit content of the blood samples, there was a reduction in the separation efficiency of these microdevices. Our best design was able to provide separation efficiency around 80% with a yield of 3% using pure blood^39^,^40^. However, extending these designs to achieve complete separation has not turned out to be trivial. We note that almost no study based only on hydrodynamic separation has demonstrated complete separation, due to difficulties noted below, attesting to the complexity of the problem involved. In this study, we present a passive, hydrodynamic microfluidic device capable of demonstrating complete separation of cells from whole blood. Almost complete separation of cells from pure blood makes the step of dilution redundant. We demonstrate through various biological tests, the high quality of plasma recovered from our microdevice. The dimensions employed in our microdevice are about hundred microns. The dimensions of this order ensure that the microdevice is free from clogging and is relatively easy to fabricate. The yield of the microdevice is in the range of 1 to 6%, depending upon the hematocrit content and the plasma channel resistance employed.

## Design principle

In the following section, we present the microdevice design and the theoretical background of design approach.

### Design of microdevice

**Figure 1:**
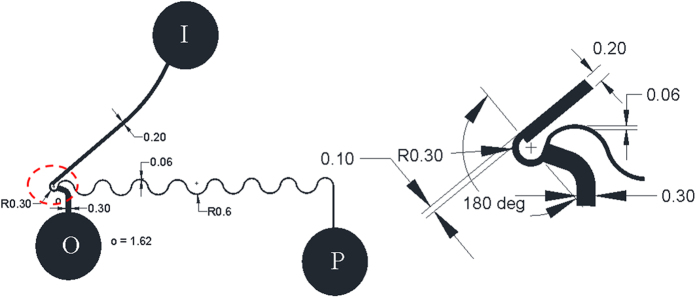
(**a**) Design of the microdevice. (**b**) Zoomed view of junction of microdevice. (I-Inlet, O-outlet, and P-Plasma reservoir) All dimensions are in mm. The design has a curvature with an inner bending radius of 0.2 mm, and an outer bending radius of 0.3 mm. Therefore, the zone of constriction has a width of 100 μm. The bend angle employed in this design is 180^0^. The microdevice have been fabricated to a depth of 60 μm.

Figure 1 provides the geometrical details of the microdevice designed in this study. The microdevice constitutes a main channel for blood inlet (I), main channel for blood outlet (O), and a plasma channel (P). The heart of the microdevice is the zone of constriction-expansion. All dimensions (in millimeter) of the microdevice are specified in Fig. 1. During the course of experiments, we tested several designs and implemented various design modifications so as to come up with a design that is capable of providing excellent separation efficiency even on pure or undiluted blood. Figure 1(a) shows the design of the microdevice. Figure 1(b) shows the zoomed view of the zone of constriction-expansion. Compared to our previous work, the present microdevice was found to be the best performer.

### Working principle

Our microdevice exploits Zweifach-Fung bifurcation law at elevated dimensions^38^,^39^,^40^ by manipulating the flow rate ratio at the bifurcation. The flow rate ratio was modified by suitably changing the resistance of the plasma and main blood outlet microchannels. It is well established in literature that Fahraeus effect is more pronounced in channels having dimensions between 21 and 221 μm^20^. The dimensions of the microdevice are in the range to exploit the Fahraeus effect. Microchannels with lower dimensions tend to clog more easily in comparison with microchannels with higher dimensions. Therefore, the smallest dimension in the microdevice was chosen so as to avoid clogging of the microchannel. This ensures that we are able to effectively exploit Fahraeus effect, and simultaneously ensure clog free operation of the microdevice.

The introduction of a bend in the design brings centrifugal effects into picture. The centrifugal force acting on cells is dependent upon the density of the cells, radius of the curvature of the bend, and velocity of the flow. The cells due to higher density are pushed towards to the outer wall of the microchannel, leaving a clear cell depleted layer along the inner wall of the microchannel. Employing an expansion zone immediately at the end of the bend results in a significant increase of the cell free region in this zone. Thus, a hybrid combination of constriction-expansion and bends provides an excellent opportunity to exploit this cell free region for further plasma extraction. Therefore, this particular design is a synthesis of various bio-physical effects and geometrical features in an optimal fashion.

### Role of inertial forces

In addition to the bio-physical and geometrical effects, inertial effects in microchannels may play an important role in separation of cells and may be utilized for manipulation of particles/cells^42^,^43^,^44^,^45^,^46^. Therefore, calculations pertaining to inertial effects in our microdevice has been made. Additional details are provided in the methods section.

Particles/cells in the flowing fluid are subjected to the lift and drag forces. The balance of these forces defines the position of the particles/cells in the microchannel^42^,^43^,^44^,^45^,^46^. DiCarlo *et al.*^44^ have shown that the ratio of relative magnitude of the lift and drag forces or *R*_*f*_ is an important factor while considering equilibrium separations and it also leads to better understanding of inertial focusing. Recently Martel and Toner^46^ have conducted a systematic study of inertial focusing in curved microchannels and found complex set of inertial focusing regimes by decoupling the effects of Reynolds number and Dean number. They showed that the particle/cells may move away or towards the center of curvature with an increase in the Reynolds number. Their research showed that for inertial focusing to occur, the confinement ratio has to be *λ* > 0.07, and Reynolds number of the particle Re_*p*_ ≥ 1. It was also shown that when *R*_*f*_  > 1lift force pushes the particles to an equilibrium position, and if *R*_*f*_ < 1 the Dean drag dominates and leads to mixing of particles/cells^44^.

The present microdevice consists of a constricted curved channel (Fig. 1). The cells (RBCs, WBCs) and platelets are suspended in plasma. Herein, we study the effect of the lift and drag forces and other important parameters such as Dean number, Reynolds number, confinement ratios and curvature ratios to evaluate the effect of inertial focusing on suspended cells. For the curved portion of the microdevice, *d*_*h*_ = 75 μm, and *R* = 250 μm. The channel Reynolds number is Re_*c*_ = 39.06, Dean number is *De* = 14.26, and curvature ratio is *δ* = 0.15. The particle Reynolds number for RBCs, WBCs and platelets (based on mean diameter of 6 μm, 10 μm and 3 μm) are Re_*p*–*rbc*_ = 0.25, Re_*p*–*wbc*_ = 0.694 and Re_*p*–*platelet*_ = 0.0625 respectively. The confinement ratios for RBCs, WBCs and platelets are *λ*_*rbc*_ = 0.08, *λ*_*wbc*_ = 0.13 and *λ*_*platelet*_ = 0.04 respectively. The ratio of lift to drag forces for RBCs, WBCs and platelets are *R*_*f*–*rbc*_ = 0.143, *R*_*f*–*wbc*_ = 0.59 and *R*_*f*–*platelet*_ = 0.016 respectively. The particle Reynolds number for all cells is less than unity, and confinement ratios for RBCs and WBCs are greater than 0.07. This suggests that there is a certain degree of inertial focusing. Further, the ratio of lift to drag forces for all the cells are calculated to be less than unity^44^. Research has also shown that inertial focusing is dependent on the concentration of cells (volume fraction), inter-particle spacing, cell properties including their shape, size and deformability. It has been further shown that the focusing behavior of cells declines with increase in concentration of the particles^45^. In the present microdevice, the particle volume fraction (Hct ~ 45%) is much above the upper limit where the inertial focusing behavior breaks down, due to inter particle interactions. Evaluating particle focusing and interaction in whole blood is highly challenging as the imaging techniques (to estimate particle focusing) are of limited use due to the presence of large number of RBCs in whole blood^47^.

### Parameters of interest

Important parameters used to quantify the plasma sample obtained from the microdevice are summarized in Table 1.

## Results and Discussion

The microdevice employed in this study has been experimentally tested on blood obtained from five different voluntary donors. Each donor had a different hematocrit content. The comprehensive quantification of the experimental data has been carried out on the blood of a donor having an inlet hematocrit of 42%. To evaluate the separation efficiency, the cell counting has been performed three times for a particular sample using a hemocytometer. The following section provides detailed analysis of the experimental results, and quantified data for the microdevice.

### Effect of flow rate and hematocrit on separation efficiency

**Figure 2:**
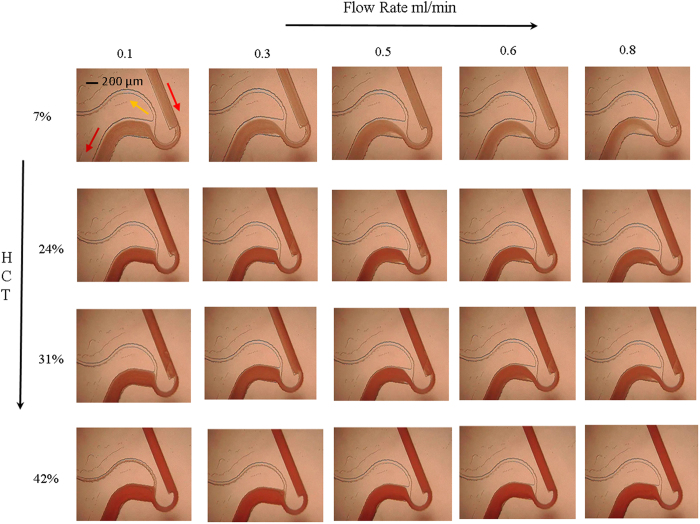
Experimental photographs of the microdevice showing the plasma separation phenomenon with variation in hematocrit (7%, 24%, 31% and 42%) and flow rates (0.1, 0.3, 0.5, 0.6 and 0.8 ml/min).

**Figure 3:**
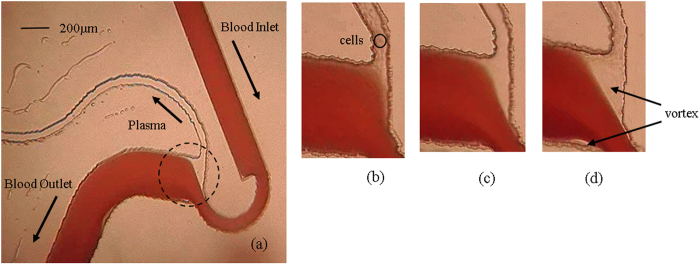
(**a**) Experimental photograph of microdevice showing plasma separation and blood flow in microchannel for pure blood (0.5 ml/min) at magnification of 4x. (**b**–**d**) Shows zoomed view of the section at 10x magnification for flow rate of 0.1, 0.5, 0.8 ml/min, respectively.

Experimental observations on microdevice at different flow rates (0.1, 0.3, 0.5, 0.6, 0.8 ml/min), and at different hematocrits (7, 24, 31, 42%) are presented in Fig. 2. This experimental image provides a comprehensive understanding of the cell free region as a function of the flow rate and hematocrit. At low hematocrit contents, it can be observed from Fig. 2 that excellent separation efficiency is achieved at flow rates as low as 0.1 ml/min. With increase of flow rate, there is an increase in the cell free region. Observe that for hematocrit 7%, irrespective of the flow rate, cells do not reconnect with the top wall of the outlet channel downstream of expansion. Therefore, there is no possibility of cells passing through the plasma channel. Quantification of the plasma sample confirmed that the separation efficiency is close to 100%. On increasing the hematocrit to 24% and 31%, a clear reduction in the cell free region is observed at low flow rates of 0.1 and 0.3 ml/min. For hematocrits of 24% and 31%, few cells tend to flow into the plasma channel at these low flow rates of 0.1 and 0.3 ml/min. However, there is a sharp increase in the cell free region on increasing the flow rate to values beyond 0.3 ml/min. That is, cells do not pass into the plasma channel, resulting in separation efficiency of close to 100% for hematocrit values of 24% and 31% at higher flow rates. With increase in the flow rate, there is an increase in the strength of vortices formed in the zone of constriction-expansion as well. At low to moderate hematocrit values (~30%), it is observed that the extent of cell free region is such that the cells are not influenced by the presence of vortex and the cells do not come into contact with the vortex. Therefore, very few cells pass through the plasma channel. Hence, there is no drastic deterioration in the values of separation efficiency and almost 100% efficiency is achieved at all flow rates for low to moderate hematocrit contents. Further experiments show that this microdevice provides excellent results even on pure or undiluted blood, having hematocrit content of 42%. An experimental video demonstrating separation for 42% hematocrit at a flow rate of 0.5 ml/min is also included as Video 1. Excellent separation efficiency values are obtained due to the higher resistance of the plasma channel (Fig. 3). For pure blood, on increasing the flow rate values beyond 0.3 ml/min, as in other cases of lower hematocrits, there is a dramatic increase in the cell free region.

However, experiments on pure blood reveal an interesting phenomenon. Figure 3 presents the magnified view of the constriction-expansion zone. This figure reveals the presence of vortex and its influence on reducing the separation efficiency. It can be observed from Fig. 3 that at low flow rate of 0.1 ml/min (Fig. 3b), cells tend to pass through the plasma channel. On increasing the flow rate to 0.5 ml/min (Fig. 3c), there is an increase in the cell free region, and cells do not appear to pass through the plasma channel. However, on further increasing the flow rate to 0.8 ml/min (Fig. 3d), the vortex increases in strength, resulting in cells passing the plasma channel and there is a reduction in separation efficiency. The above experimental investigations on pure blood reveal that on increasing the flow rate, there is an increase in the cell free region. But, due to the simultaneous increase in the strength of the vortex, there is a reduction in the separation efficiency.

**Figure 4:**
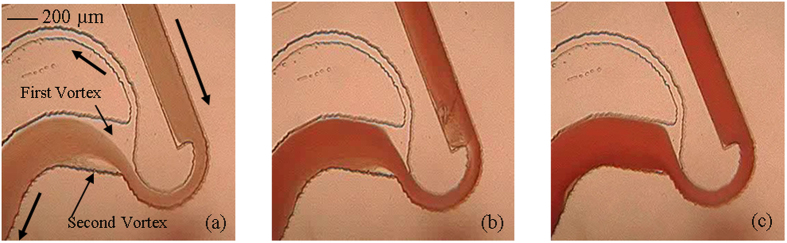
Experimental photograph of vortex formation at the junction of microdevice at a flow rate of 0.5 ml/min at Hct of (**a**) 7% (**b**) 24% (**c**) 42%. The presence of this second vortex with high strength can be clearly observed at lower hematocrits.

An additional observation in this design is regarding the presence of a second vortex. Due to the high radius of curvature in this design, a second vortex, with strength proportional to the flow rate, is observed at flow rates higher than 0.1 ml/min. It is observed in the cases with hematocrit 24% and 42% (flow rate 0.5 ml/min), there is a reduction in the strength of this second vortex, shown in Fig. 4. As it can be clearly observed that (especially at low hematocrits) the second vortex region is relatively free from presence of blood cells. Therefore, the second vortex can potentially be exploited by including another plasma channel at this zone, thereby resulting in further increment of plasma collected from the microdevice.

**Figure 5:**
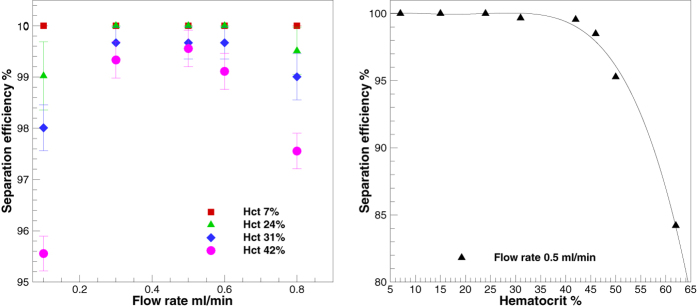
(**a**) Separation efficiency versus flow rate for different hematocrit values. (**b**) Experimental data and data curve fit for separation efficiency versus hematocrit at flow rate of 0.5 ml/min. At low hematocrit content of 7%, the separation efficiency is almost 100% at all the flow rates considered. For Hct content of 24, 31 and 42%, there is some drop in the separation efficiency at low flow rate of 0.1 ml/min and at higher flow rate of 0.8 ml/min. At flow rate of 0.5 ml/min, for Hct 31% separation efficiency of 99.66 ± 0.31% is obtained and with undiluted blood, separation efficiency of 99.55 ± 0.35% is obtained.

Figure 5(a) presents the quantified results of the separation efficiency obtained on different hematocrit values, at different flow rates. During the course of our experiments, we noted that the time taken to attain steady- state is about 20 to 25 seconds. This value suggests that the steady-state is reached very quickly. Once steady-state is reached, the microdevice begins to demonstrate excellent separation efficiency. It can be clearly seen that excellent separation efficiencies, irrespective of the hematocrit content, are obtained in the flow rate range of 0.3–0.5 ml/min. The influence of vortices for the drop in separation efficiency at higher flow rates has already been discussed. With pure or undiluted blood, we obtain a separation efficiency of 99.55 ± 0.35% at a flow rate of 0.5 ml/min. Depending upon the hematocrit employed, and by varying the plasma channel resistance, the yield obtained in this particular design at flow rate of 0.5 ml/min is about 1 to 6%. The yield can be improved further by employing the ideas proposed by Prabhakar *et al.*^39^. Inertial based techniques for separation have shown promising values of yield. However, these techniques require dilution of blood. A comparative values of yield and separation efficiency obtained (for pure and diluted blood) in various inertial based techniques has been provided in Table 2.

### Flow cytometry data

**Figure 6:**
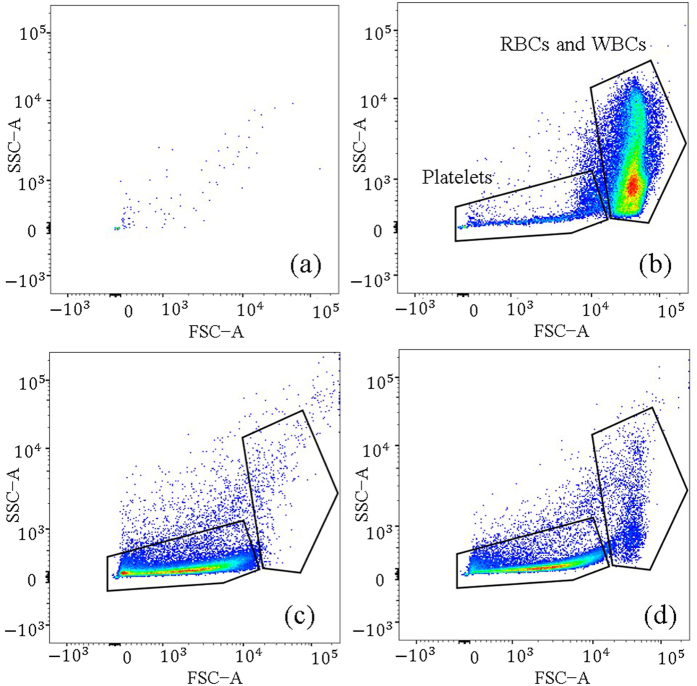
Flow cytometry results of various samples. (**a**) Negative control (filtered PBS). (**b**) Positive control (whole blood). (**c**) Centrifuged plasma sample. (**d**) Plasma separated from whole blood using the present microdevice.

Flow cytometry study has been carried out as this analysis gives an in-depth information about the constituents of the sample and also the cell viability. Figure 6 shows the flow cytometric data (50,000 events) obtained for negative control, positive control, centrifuged plasma, and plasma separated using our microdevice. The horizontal axis is the Forward scatter (FSC), and is a measure of the cell size. The vertical axis is the Side scatter (SSC), and is a measure of the cell granularity. Figure 6a shows the flow cytometric data of the negative control, i.e., ultra-pure filtered PBS (Phosphate-buffered saline). As expected, this sample is free from any cells.

Figure 6b shows the flow cytometric data obtained with pure blood. We consider it to be the positive control, as red blood cells, white blood cells, and platelets will be present in this sample. It can be interpreted from this figure that the platelets (and cell debris), due to their low cell size and granularity, occupy the bottom left of the figure. The cells (RBCs + WBCs) have a higher granularity and size, and therefore occupy a large region in the top right portion of the figure. There is a smooth transition from the bottom left to the top right. The intermediate region can be either platelets or cells^37^.

Figure 6d shows the flow cytometric data for the plasma sample obtained from pure blood using our microdevice. On comparison with Fig. 6a, it can be noted that the count of RBCs and WBCs in Fig. 6b is significantly reduced, but the count of platelets (and cell debris) has increased. This suggests that for 50,000 events of plasma sample, large number of events represent those of platelets (and cell debris), implying that the microdevice has been able to effectively separate the blood cells. Also, the data scatter between Fig. 6b,d is minimal, thereby revealing excellent cell (sample) viability^37^,^44^. The slight scatter of data can be either small RBCs or cell debris. Thus it can be interpreted that the plasma sample is relatively free from hemolysis.

Figure 6c shows the data from the centrifuged plasma (3500 rpm for 15 min). Even in this figure, the highlighted portion in the top right quadrant is almost devoid of cells. This is expected as the centrifuged plasma has to be free from blood cells. Interestingly, in centrifuged plasma for our experimental conditions, we observe platelets. On comparison with Fig. 6d, we observe that barring a very small region on the top right, Fig. 6c,d are almost identical. This implies that our microdevice is able to separate cells with a separation efficiency close to that of a centrifuged sample. However, due to the presence of few blood cells in the separated plasma (from our microdevice), the separation efficiency of our microdevice is slightly lower than that of the centrifuged sample. It is to be noted that platelets are visible in both the centrifuged plasma and the plasma from our microdevice, and their total count in both these samples is almost equivalent. Therefore, purity of the plasma obtained from our sample is almost equivalent to that of centrifuged plasma. The RBCs and WBCs were counted using a hemocytometer^48^. For the inlet blood sample having ~4.5 million RBCs/μL and ~7900 WBCs/μL, the separated plasma had ~20,000 RBCs/μL and ~80 WBCs/μL (with an uncertainty in measurement of about 5%).

### Proposed empirical equation

In this section, we present a data fit to the experimental curve obtained at a flow rate of 0.5 ml/min, shown in Fig. 5b. It is suggested to operate the microdevice at a flow rate close to 0.5 ml/min, because of the high value of separation efficiency at this flow rate. The following piecewise second order curve is seen to fit the experimental data


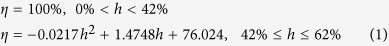


where, ‘*η*’ is the separation efficiency in percentage, and ‘*h*’ is the percentage of hematocrit. The goodness of fit value is R^2^ = 0.9975. The proposed equation is valid for flow rate of 0.5 ml/min in the hematocrit range of 42 to 62%. The proposed equation suggests a separation efficiency of 99.68% at 42% Hct, and separation efficiency of 84% at 62% Hct. The proposed equation is useful to get an idea about the dependency of separation efficiency on hematocrit, and may be useful for modelers^49^. It is especially helpful in situations of higher hematocrit where it is challenging to carry out experiments. For example, on extrapolating the validity of equation to much higher hematocrit, say 70% Hct, the predicted performance of the microdevice in terms of separation efficiency is 72.9%.

## Highlighting features of the developed microdevice

In this section, we bring out several unique features of the developed microdevice. We also provide experimental demonstrations of the other major advantages of the proposed microdevice.

### Clog free operation

Formation of aggregates (clots) is a very common occurrence in blood, and has been widely reported by researchers^4^,^5^,^37^. Approaches to blood plasma separation at micro-scale, such as sedimentation and filtration, although have promise in providing good separation efficiency have not been very successful due to clogging of the microdevice (due to formation of clots)^6^. In our experience, some of the microdevices based on hydrodynamic principles, reporting excellent separation efficiency on pure blood, are not totally viable as they tend to clog within the first few experiments. Such observations have been reported by other researchers as well^37^. This is primarily due to the low microchannel dimensions employed in the earlier designs, making those designs prone to clogging. This highlights that clogging is a major challenge in design of such microdevices^4^,^5^. In the present microdevice, the minimum width and height of the passages through which blood flows are 100 and 60 μm respectively (for comparison, the size of an RBC is 6–9 μm). The employed dimensions are significantly higher than that of the designs presented in literature, which are capable of achieving good separation efficiency on whole blood.

**Figure 7:**
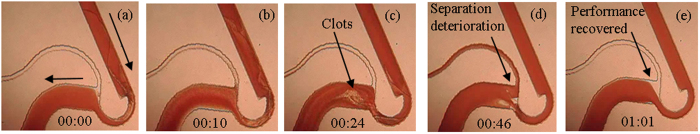
Demonstration of clog-free operation. Images taken at time (**a**) 0, (**b**) 10, (**c**) 24, (**d**) 46 and (**e**) 61 seconds. Notice that the cell aggregates are cleared in less than a minute of operation. The presence of aggregate in the microchannel alters the flow behavior (see Fig. 7 **b**–**d**). This results in flowing of large number of cells in the plasma channel, and there is a decrease in the separation efficiency during this time period.

In our study, enhanced dimensions of the microchannels coupled with the moderate flow rates employed ensure operation of the microdevice in a continuous clog free manner. To experimentally demonstrate these aspects, we employed blood with increased clots. The complete video of the clog free operation of the microdevice is provided as Video 2. A few experimental images taken on whole blood at a flow rate of 0.5 ml/min are provided in this section. Figure 7 shows the progression of clog in the microchannel with time. It can be clearly observed from Fig. 7(a–c) that the microdevice clears the aggregations and the functioning is restored to the original state of excellent separation in about a minute. Therefore, this microdevice is capable of removing the aggregates which are normally encountered, and provides a clog free operation. The word ‘clog-free’ has been used to suggest that the microdevice would clear aggregates (whose size is lower than channel dimensions), and thereby restore the initial working conditions. If the microdevice is not capable of removing these blood aggregates, the device tends to clog, thereby resulting in failure of the microdevice.

It is to be noted that if the size of the aggregate is significantly larger than our microchannel dimensions, there is a chance for this particular microdevice to fail. However, in our experience, we have noticed that it is rare to encounter an aggregate which is sufficiently larger than our microchannel dimensions. It is very difficult to predict the frequency (and size) of aggregate occurrence, as it is bears a complex dependency on various parameters (both biological and in terms of blood handling). Nevertheless, we can say that the frequency of clogging is dependent on the inlet blood conditions. Therefore, the maximum working duration of the microdevice is a strong function of the quality of the blood at microdevice inlet.

### Experiments on enhanced hematocrit contents

An additional exercise of experimenting with blood having very high hematocrit contents was carried out. The excellent performance of the microdevice for pure and diluted blood samples has already been demonstrated. However, in practice, sometimes situations may arise in which the microdevice must possess the capability to separate plasma from individuals with very high hematocrit than those normally encountered. Therefore, this study on high hematocrit (upto 62%) provides us with an understanding about the feasibility of the proposed microdevice for individuals with polycythemia. Polycythemia (also known as polycythaemia or polyglobulia) is a disease state in which hematocrit level of blood increases. A high hematocrit level, greater than 55% is seen in polycythemia. It can be due to reduction in the volume of plasma (“relative polycythemia”) or an augmentation in the number of red blood cells (“absolute polycythemia”).

For the purpose of these experiments, the blood sample was allowed to naturally sediment. Thereafter, some plasma volume was removed from the blood sample. Therefore, the hematocrit content of the blood sample was enhanced by plasma removal. Higher hematocrit range of 46, 50 and 62% were chosen for the purpose of these experiments. In the next stage of experimentation, we tried to assess the performance of our microdevice, and bring forth the separation capability of our microdevice at these very high hematocrit contents. Experimental video for elevated hematocrit of 62% is presented as Video 3.

**Figure 8:**
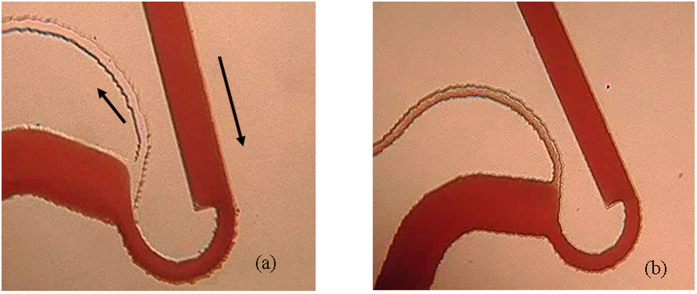
(**a**) Experimental image for microdevice at a flow rate of 0.5 ml/min with (**a**) Hct 46% and (**b**) Hct 62%.

Figure 8(a) presents an experimental image at 0.5 ml/min for hematocrit of 46%. It can be observed that, even at this hematocrit, there was not much reduction in the cell free region. The separation efficiency value for 46% hematocrit at 0.5 ml/min is about 98.5%. Experiments at 50% hematocrit revealed some decrease in the cell free region. However, even at this hematocrit, the separation efficiency obtained is as high as 95.3%. The final experiment was carried out at 62% hematocrit at a flow rate of 0.5 ml/min. The corresponding experimental image is presented in Fig. 8(b). At this high level of hematocrit, there is no truly visible cell free region. However, the outer region of the plasma channel has only a few cells flowing through it. Nevertheless, in comparison to the experimental images carried out at hematocrits lower than 62%, we can observe that the number of cells flowing through the plasma channel is higher. Given, the substantial increase in the hematocrit content, this was an expected outcome. The separation efficiency obtained is reasonably high at 84.2%.

Also, an additional objective of this study is to bring forth the advantage of our microdevice in situations where recirculation of blood sample is feasible. On plasma getting separated, theoretically, it is quite obvious that the hematocrit level of the sample from blood outlet reservoir increases (in comparison to inlet blood samples). With successful separation of plasma with such high hematocrit (46–62% Hct) content blood, it is highly possible that even if we recirculate blood, i.e. from blood outlet reservoir to blood inlet reservoir of device, the device will still be able to separate plasma efficiently till the inlet hematocrit value reaches to about 60%. Thus employing blood recirculation approach (between blood inlet and blood outlet reservoir), we may increase the quantity of plasma recovered (yield) from a given quantity of inlet blood sample.

Therefore, these experiments at artificially enhanced hematocrit contents reaffirm the excellent separation capability coupled with clog free operation of the microdevice presented in this study. The above results prove that blood plasma separation for individuals with polycythemia (Hct > 55%), normal (40–45% Hct) and anaemic patients is possible with the present microdevice. Therefore, irrespective of hematocrit content, our microdevice is able to separate plasma with high separation efficiency, while ensuring clog free operation. This will pave the way to develop a versatile micro-total analysis system for detection of blood analytes.

### Summary of the microdevice

The significant advantages of our microdevice are in terms of obtaining excellent separation efficiency for the entire range of hematocrit, i.e. from diluted to undiluted (including enhanced hematocrits) blood; almost 100% percent separation efficiency with pure blood, thereby reduces an additional step of dilution; small surface area of the microdevice (~2 cm^2^); high plasma extraction rate; obtaining decent amount of plasma (yield of 1 to 6%); continuous clog free operation; hemolysis free operation; ensuring that high-quality plasma is obtained from the microdevice in order to carry out the subsequent steps of analyte detection.

It is noted that our microdevice works effectively in the flow rate of 0.3 to 0.5 ml/min. The rate of plasma extraction is about 5 to 30 μL/min for an inlet flow rate of 0.5 ml/min. This rate of plasma extraction is significantly higher than other microdevices presented in the literature, where typically flow rate of the order μL/hr is employed.

## Biological Validation

In general, the ultimate aim of plasma separated from blood is detection of biomarkers or analytes. In order to demonstrate this conclusively, biological validation of the separated plasma is imperative. Depending on the analyte of interest, one can perform wide variety of tests on the separated plasma. However, owing to practical limitations, it is not feasible to carry out every possible test on the separated plasma. In this regard, to test the viability of our plasma sample, we have undertaken scientific tests which are very common. Large population in the world suffers from diabetic related problems. Also, pregnancy is a common occurrence. Therefore, we have chosen to detect glucose (indicator of diabetes), and hCG (human chorionic gonadotropin) hormone (indicator of pregnancy) from our plasma sample. Additionally, shear stress values were evaluated to assess hemolysis. Proteins are vital indicators of the performance of human body. Therefore, ‘Total protein detection’ in the plasma sample was carried out by UV spectrophotometric analysis. We believe that these tests, though limited in number, would provide a better understanding of the feasibility of plasma sample obtained from our microdevice to carry out other diagnostic tests.

### Assessment of hemolysis

Various researchers have proposed the threshold shear stress value to be 452 Pa^50^,^51^. In our microdevice, the maximum shear stress value obtained for pure blood in the flow rate range of 0.1 to 0.5 ml/min is estimated to be between 30 to 152 Pa (see Methods section), thereby confirming that the shear stress in our design is lower than the threshold value for hemolysis.

### Detection of Blood Plasma Protein using UV spectrophotometric method

After the on-chip blood plasma separation process, UV spectrophotometric analysis was performed to detect the presence of proteins in a sample of blood plasma extracted from pure blood using our microdevice. The direct UV spectrophotometer method was employed for this purpose. This method of protein detection has number of advantages over traditional calorimetric assay^52^. Quantification of the protein from desired sample is obtained by directly measuring absorbance, as the former is fast, easy, robust and convenient.

**Figure 9:**
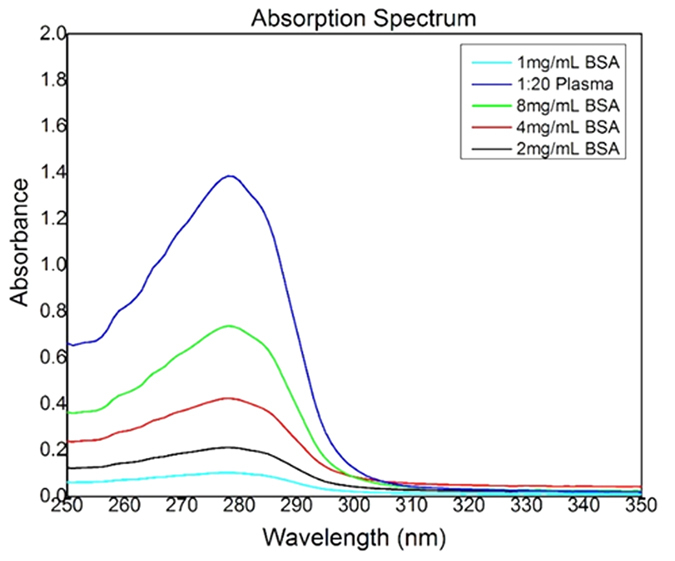
Graph of protein absorbance versus wavelength.

The absorption of protein in the ultraviolet region of 250 nm to 300 nm is commonly related to the presence of the aromatic ring in the protein molecule. The absorbance of a sample is proportional to the number of absorbing molecules in the spectrophotometer light beam. The aromatic amino acids tryptophan (Trp) and tyrosine (Tyr) present in protein exhibits the absorption maxima between 275 to 280 nm because of the delocalized electrons in aromatic systems^53^,^54^. As shown in Fig. 9, UV-Vis absorption peak of test solution at 280 nm has an absorbance value of around 0.7 (well within significance range). Thus, we could detect proteins from the plasma sample obtained from our microdevice.

### Detection of Random blood Glucose levels

A simple and common test carried out in diagnostics is the measurement of glucose levels in blood. This test can also serve as an effective indicator of the plasma quality obtained from our microdevice. Table 3 presents the values obtained for plasma samples from three different individuals. R_1_ to R_4_ are the glucose values of positive control (undiluted blood). G_1_ to G_4_ are the glucose values of plasma sample obtained from whole blood using our microdevice. According to the manufacturer of test kit, the glucose content is detected within an error of ±5%, the mean imprecision being less than 3%, with a coefficient of variation less than 2.1%. Within these experimental uncertainties, the glucose values obtained from our blood plasma sample are in good agreement with the values which are normally encountered in random blood glucose tests^55^.

### Detection of hCG (Human chorionic gonadotropin) hormone

As a part of our analyte detection, we chose to detect hCG hormone from the plasma obtained from our microdevice. We opted to detect hCG hormone, as it serves as an excellent indicator of pregnancy. We carried out experiments by enhancing hCG levels in the blood inlet.

Table 4 presents the quantified results of the experiments. As expected, due to the absence of hCG hormone in the negative control, i.e., ultra-pure water, the test kit shows a negative result. For the positive control (hCG Injection 10000 IU), the test kit shows positive. As the hCG concentration in plasma sample H_1_ is below the detection limit (for pregnancy), the test kit indicates a negative result. For other samples, i.e., H_2_, H_3_ and H_4_, the hCG concentration levels in the plasma are above the detection limits (for pregnancy), and yield a positive result. This confirms that the plasma obtained from our microdevice can be successfully used to determine the presence or absence of pregnancy in an individual. With this experiment we concluded that during the plasma separation process, concentration of analytes of interest remain stable.

## Conclusions

In this study, we developed a microdevice exhibiting plasma separation from undiluted blood in an effective and efficient manner. The final microdevice presented is a synthesis of various bio-physical and geometrical effects, and is an outcome of experimental and theoretical understanding of the basic laws governing the flow behavior of blood in small passages (microchannels). The microdevice proposed in this study addresses the challenges associated with experimenting on undiluted blood. The design presented offers simplicity in fabrication, continuous clog free operation and excellent recovery of high quality plasma. Additional advantage is with respect to dimensions of the microchannel, which are high enough to avoid clogging by blood cells, an issue which is normally encountered in microdevices operating on high hematocrit blood.

A separation efficiency of ~99.5% has been achieved on pure/undiluted blood. A comprehensive experimental study has been carried at different hematocrits and flow rates. This microdevice shows excellent separation efficiency in the flow rate range of 0.3–0.5 ml/min and at all hematocrit values (diluted to pure blood). Experiments on extremely high hematocrit (upto 62%) contents also revealed excellent separation capability of the microdevice. Additionally, we had carried out flow cytometric measurements on the separated plasma. Further, the plasma obtained is from undiluted blood. Therefore, there is no scope for the loss of target analytes which are to be detected in the separated plasma. An experimental validation of this assertion is carried out by biologically characterizing the plasma sample viability by various methods. Citing the above factors of best separation efficiency on whole blood, decent yield, elevated microchannel dimensions, clog free operation, minimal time requirement for sample collection with decent yield, hemolysis free sample, small surface area, simple fabrication techniques; we believe that the microdevice presented in this study possesses all the features required from the practical point of view to realize a μ-TAS (micro total analysis system). This plasma separation device may be effectively used in μ-TAS for extracting and presenting plasma to integrated microfluidic biosensor unit (designed for sensing any analyte of interest present in blood).

## Methods

The following section provides a brief description of fabrication techniques employed, the experimental procedure and certain important definitions which are adopted to quantify the microdevice; additional details are available elsewhere^38^,^39^,^40^. All the blood samples in this study were obtained with written informed consent from all subjects. The blood samples were pretested for any communicable diseases. All the experiments in this study were performed in accordance with the guidelines and regulations which were approved by the bio-safety committee, Indian Institute of Technology Bombay, India.

### Fabrication of microchannels

The microdevices employed in this study were fabricated using the process of conventional lithography. Here, we present a brief description of the fabrication stages. As a first step, microchannels on silicon wafers were fabricated using SU8–2050 photoresist by employing the technique of photo lithography. The fabricated microchannels had a depth of 59.82 ± 1 μm. Thereafter, the process of soft lithography was employed to fabricate the microchannels using the widely used polymer PDMS (Polydimethylsiloxane). Mixture of PDMS and curing agent in ratio of 10:1 (w/w) was prepared and poured over SU-8 mold with the design. This was followed by curing in an oven at a temperature of 70 °C for about an hour. In the next step, PDMS which now has the intended design engraved on it is peeled off from the SU-8 mold. Holes for the reservoirs were punched at appropriate locations to facilitate fluid flow through microchannels. The final step in the fabrication process was to bond the PDMS structure (with design) on to the glass slide having a thin layer (of few microns) of semi-cured PDMS (ratio of PDMS and curing agent for this thin layer is 6:1). This resulted in the final ready to use PDMS based microdevice.

### Experimental setup

The experimental setup involves a syringe pump (Cole-Parmer) to enable blood flow in the microchannels, microfluidic tubing, connectors, and microdevice. CCD camera attached to the Microscope (Olympus CH 20i) was used to capture experimental images. Freshly drawn human blood from healthy voluntary donors was obtained. Blood was mixed with anti-coagulant EDTA. In case of diluted blood samples, normal saline (0.9% Sodium Chloride) was used as a diluting agent, and was added in appropriate amounts to obtain blood samples with varying hematocrit contents. Hematocrit values in the range of 7 to 62% were adopted in this study. All the experiments were carried out within 3 hours of blood collection. The flow rates employed in this study ranged from 0.1 to 0.8 ml/min. Hemocytometer (Neubauer chamber) was employed to quantify the plasma samples obtained in our experiments.

### Flow cytometry

Flow cytometry experiments were carried out on a BD FACS Aria Special Order Research Product (SORP) system (BD Biosciences, San Jose, USA). The data acquisition software was BD FACS Diva 6.1.3. The data obtained was analyzed by FLOWJO software. An unstained sample would suffice the requirements for a qualitative estimate using flow cytometer. However, the quantitative measurement of separation efficiency is not carried out using flow cytometry data. The complete quantification of separated plasma is carried out using a hemocytometer. Negative control in these measurements was ultra-pure filtered PBS, and Positive control was pure blood. The samples tested were centrifuged plasma, and plasma obtained from pure blood using our microdevice. On each sample, the data for 50,000 events was recorded.

### Inertial focusing

The lift forces are responsible for lateral migration of the particles/cells across the flow streamlines and consists of two components: the wall interaction lift force (due to particle interaction, directed away from the channel wall) and shear gradient lift force (due to the parabolic velocity profile, directed towards the channel walls). The net lift force is the superposition of these two components of lift forces. The net lift force can be defined as 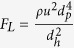, where *d*_*p*_ is the particle diameter, *u* is the average fluid velocity, *ρ* is the density of the fluid, and *d*_*h*_ is the hydraulic diameter of the microchannel^56^. In addition to the lift forces, presence of a curvature in the geometry generates secondary flows in a microchannel. Therefore, an additional drag force, named as Dean drag is introduced. Due to the secondary flows counter rotation vortices are set up in the microchannel (top and bottom of the channel i.e. perpendicular to the stream wise direction). The strength of the secondary flow can be related to a dimensionless number called Dean Number (*De*). The Dean number is defined as 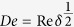, where 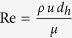 is the Reynolds number and *δ* is the curvature ratio, defined as (*δ* = (*d*_*h*_)/(2*R*))^42^,^43^,^44^,^45^,^46^. In this definition, *d*_*h*_ is the hydraulic diameter, defined as 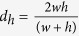, where ‘*w*’ is the width of microchannel, ‘*h*’ is the height of microchannel, and ‘R’ is the mean radius of curvature. The Dean drag force can be defined as *F*_*D*_ = 3*πμU*_*D*_*d*_*p*_, where *μ* is the viscosity of the fluid, and U_D_ is the average Dean velocity (*U*_*D*_ = 1.8 × 10^−4^*De*^1.63^)^43^,^57^,^58^. To study the effects of inertial focusing on cells/particles, Reynolds number of the particle is an important parameter, and it is defined as 
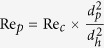
, where Re_c_ is the channel Reynolds number, defined as 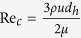. Two other important parameters are required to assess the role of inertial effects (focusing) are: the particle confinement ratio 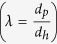, and the ratio of lift to drag force, 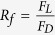
44,45,46,59.

### Shear stress calculation

The flow in our microdevice is laminar. In laminar flow, the friction factor (f) is a function of Reynolds number only, and the roughness of the microchannel has no role to play in the friction factor values. The shear stress in our microchannel was evaluated based on the dimensions of the inlet microchannel. Our inlet microchannel has height of 60 μm, and a width of 200 μm. This corresponds to an aspect ratio of 3.34. For a channel with rectangular cross section, having this particular aspect ratio, the friction factor can be approximated as^60^ 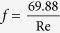. For a non- circular pipe, 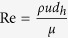, where Re is the Reynolds number, ρ is the density of fluid (blood), *u* is the average flow velocity, *d*_*h*_ is the hydraulic diameter, and *μ* is the dynamic viscosity (blood). The relation between the friction factor and wall shear stress is^60^ 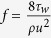. Substituting the corresponding values, gives us the estimate of approximate shear stress on the cells.

### Detection of Blood Plasma Proteins

Determination of protein requires standard curve for comparison, in order to determine unknown concentration of proteins in sample on basis of observed absorbance value. Milli-Q water was used as the negative control in these experiments. BSA (Bovine Serum Albumin) (Sigma Aldrich) solutions were prepared in milli-Q water for standard curve preparation. The spectrophotometric (Perkin Elmer) instrument has been used for the detection of protein. The spectrophotometric method was used for the detection of protein as described by Soedjak *et al.*^61^. Standard curve (positive control) was prepared by preparing BSA stock solution with concentration 8 mg/mL, and serially diluted it at 1:1, 1:2 and 1:4 ratios to obtain 4 mg/mL, 2 mg/mL and 1 mg/mL dilutions, respectively. Thus, a calibration curve with BSA concentrations 1 mg/ml, 2 mg/ml, 4 mg/ and 8 mg/ml was obtained.

Blood plasma obtained from the microdevice using whole blood sample was collected in Eppendorf tubes. For detection of presence of protein contents in plasma, plasma sample was diluted with milli-Q water (1:20) in order to obtain its absorbance in 0.1 to 1 value range (range of absorbance significance) of BSA standard curve data. For the first reading, 1:10 ratio plasma sample solution was prepared, a peak was absorbed at 280 nm but some noise appeared with absorbance value at around 3, hence the reading was insignificant. So for the second reading, 1:20 ratio sample was analyzed.

### Random blood glucose test

To detect the Random blood Glucose levels, commercially available ACCU-CHEK Active, Blood Glucose monitory system, manufactured by Roche Diagnostics GmbH was employed. This detection system is based on hexokinase method, and is calibrated using the ID-GCMS (Isotope Dilution Gas Chromatography Mass Spectrometry) method. It is to be noted that the test kit is intended to work with whole blood. However, glucose levels can also be detected from the plasma, and these values are about 10 to 12% higher than the values obtained from whole blood^55^. For these experiments, the negative control chosen was ultra-pure water. As a positive control, we had chosen the whole blood sample. The samples from two different individuals were evaluated for the purpose of this study. Sample refers to the plasma from undiluted blood using our microdevice. In order to prevent glycolysis, the experiments for this study were conducted within the first half an hour of blood collection.

### Detection of hCG

An hCG level of less than 5 mIU/ml is considered negative for pregnancy, and anything above 20 mIU/ml can be considered positive for pregnancy^62^. Because of easy and wide availability of hCG test cassettes (used for pregnancy detection), we employed it in our experiments. In general, difference between plasma and urine is the plasma protein (present in plasma and not in urine), which may not cause any interference with the testing^63^. Therefore, we realized that the test kit which is designed for urine sample, can also handle plasma (and is experimentally demonstrated in our study).

We expected to find positive hCG results in plasma (extracted with present device) of expected pregnant human subjects (within 10 days of conception). Initially, we achieved positive results with such (pregnant or expecting pregnancy) human subjects. However, to obtain samples regularly from such human subjects for our experiments became unviable. So, as an alternative plan, we mixed standard concentration of hCG injection (commercially available as HUCOG) in blood to achieve the desired concentration.

For these experiments, negative control was ultra-pure water. As a positive control, we used the sample from hCG Injection 10000 IU. Different samples with varying concentration of hCG were prepared. Initially, we mixed hCG Injection 10000 IU powder in 1 litre of normal saline (0.9% Sodium Chloride). Afterwards, we mixed 1 ml of this hCG solution with 100 ml of normal saline. This would lead to ~100 mIU/ml of hCG concentration. Subsequently, 1 ml of this sample was mixed with 6 ml of whole blood, from which plasma was collected through our microfluidic device. In a similar manner, by altering the volumes of hCG solution and saline water, different blood samples with varying hCG concentrations of about 5, 50, 75 and 100 mIU/ml were prepared. Subsequently, plasma sample was tested for presence of hCG with a hCG test cassettes (commercially available as *i-can* test cassette, PIRAMAL Healthcare).

## Additional Information

**How to cite this article**: Tripathi, S. *et al.* Microdevice for plasma separation from whole human blood using bio-physical and geometrical effects. *Sci. Rep.* **6**, 26749; doi: 10.1038/srep26749 (2016).

## Supplementary Material

Supplementary Information

Supplementary Video 1

Supplementary Video 2

Supplementary Video 3

## Figures and Tables

**Table 1:** Definitions of parameters adopted to quantify separation results.

Parameter	Definition	Interpretation	Expression	Symbol
Separation Efficiency	Percentage of cells removed from inlet blood sample	Purity of plasma	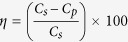	*C*_*s*_ is the number of cells per μL of blood at the inlet of the main blood inlet channel, *C*_*p*_ is the number of cells per μL of plasma sample at the outlet of the plasma channel
Yield	Ratio of extracted Plasma volume to inlet volume of blood	Percentage of plasma extracted	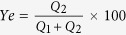	*Q*_1_ is flow rate of the main blood outlet channel*Q*_2_ is flow rate of plasma outlet microchannel
Flow rate ratio	Ratio of bifurcating channel flow rates	Approximation of resistance and yield of microchannel	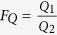	

**Table 2:** Comparison among few microdevices based on passive separation techniques.

Group	Hct%	Efficiency %	Yield %
Lee *et al.*^42^	45	60	62.2
Nivedita *et al.*^43^	0.45	90	17
Blattert *et al.*^32^	45	58	5–10
Mach *et al.*^64^	0.5% v/v dilution	100	–
Kerhoas *et al.*^37^	45	99	5
Sollier *et al.*^65^	1:20 dilution	99	10.7
Present work	42	99.5	1–6

**Table 3:** Controls and samples used for Random blood glucose test.

Individual	Negative control(mg/dL)	Positive control (mg/dL)					Plasma sample (mg/dL)				
		R_1_	R_2_	R_3_	R_4_	Mean/StandardDeviation	G_1_	G_2_	G_3_	G_4_	Mean/StandardDeviation
#1	0	102	100	101	103	101.5/1.29	109	107	109	111	109/1.63
#2	0	74	76	75	76	75.25/0.95	86	85	87	88	86.5/1.29
#3	0	82	81	80	82	81.25/0.95	91	93	89	92	91.25/1.70

**Table 4:** Controls and samples used for hCG test.

Sample	Expectedoutcome	Actualoutcome
Negative control (ultra-pure water)	Negative	Negative
Positive control (hCG Injection 10000 IU)	Positive	Positive
H_1_ (5 mIU/ml)	Negative	Negative
H_2_ (50 mIU/ml)	Positive	Positive
H_3_ (75 mIU/ml)	Positive	Positive
H_4_ (100 mIU/ml)	Positive	Positive

## References

[b1] Fung Y. C. Biomechanics: mechanical properties of living tissues. 2nd edn. Springer Science & Business Media (2013).

[b2] Caro C. G. The mechanics of the circulation. 2nd edn. Cambridge University Press (2011).

[b3] Kersaudy-Kerhoas M. & Sollier E. Micro-scale blood plasma separation: from acoustophoresis to egg-beaters. Lab Chip. 13, 3323–3346 (2013). 23824514 10.1039/c3lc50432h

[b4] Sollier E., Rostaing H., Pouteau P., Fouillet Y. & Achard J. L. Passive microfluidic devices for plasma extraction from whole human blood. Sens. Actuators, B. 141, 617–624 (2009). 10.1109/IEMBS.2009.5333314 19964193

[b5] Tripathi S., Kumar Y. V. B. V., Prabhakar A., Joshi S. S. & Agrawal A. Passive blood plasma separation at the microscale: a review of design principles and microdevices. J. Micromech. Microeng. 25, 083001 (2015).

[b6] Pamme N. Continuous flow separations in microfluidic devices. Lab Chip. 7, 1644–1659 (2007). 18030382 10.1039/b712784g

[b7] Lenshof A. & Laurell T. Continuous separation of cells and particles in microfluidic systems. Chem. Soc. Rev. 39, 1203–1217 (2010). 20179832 10.1039/b915999c

[b8] Nakashima Y., Hata S. & Yasuda T. Blood plasma separation and extraction from a minute amount of blood using dielectrophoretic and capillary forces. Sens. Actuators, B. 145, 561–569 (2010).

[b9] Laurell T., Petersson F. & Nilsson A. Chip integrated strategies for acoustic separation and manipulation of cells and particles. Chem. Soc. Rev. 36, 492–506 (2007). 17325788 10.1039/b601326k

[b10] MacDonald M. P., Spalding G. C. & Dholakia K. Microfluidic sorting in an optical lattice. Nature. 426, 421–424 (2003). 14647376 10.1038/nature02144

[b11] Huh D. *et al.* Gravity-driven microfluidic particle sorting device with hydrodynamic separation amplification. Anal. Chem. 79, 1369–1376 (2007). 17297936 10.1021/ac061542n PMC2527745

[b12] Lee B. S. *et al.* A fully automated immunoassay from whole blood on a disc. Lab Chip. 9, 1548–1555 (2009). 19458861 10.1039/b820321k

[b13] Jung J. & Han K. H. Lateral-driven continuous magnetophoretic separation of blood cells. Appl. Phys. Lett. 93, 223902 (2008).

[b14] Zhang X. B. *et al.* Gravitational sedimentation induced blood delamination for continuous plasma separation on a microfluidics chip. Anal. Chem. 84, 3780–3786 (2012). 22449121 10.1021/ac3003616

[b15] Inglis D. W., Davis J. A., Austin R. H. & Sturm J. C. Critical particle size for fractionation by deterministic lateral displacement. Lab Chip. 6, 655–658 (2006). 16652181 10.1039/b515371a

[b16] Moorthy J. & Beebe D. J. *In situ* fabricated porous filters for microsystems. Lab Chip. 3, 62–66 (2003). 15100783 10.1039/b300450c

[b17] Chen X., Cui D., Liu C., Li H. & Chen J. Continuous flow microfluidic device for cell separation, cell lysis and DNA purification. Anal. Chim. Acta. 584, 237–243 (2007). 17386610 10.1016/j.aca.2006.11.057

[b18] Fahraeus R. The suspension stability of the blood. Physiol. Rev. 9, 241–274 (1929).

[b19] Fåhræus R. & Lindqvist T. The viscosity of the blood in narrow capillary tubes. Am. J. Physiol.-legacy content 96, 562–568 (1931).

[b20] Barbee J. H. & Cokelet G. R. The Fahraeus effect. Microvasc. Res. 3, 6–16 (1971). 5092929 10.1016/0026-2862(71)90002-1

[b21] Cokelet G. R. & Goldsmith H. L. Decreased hydrodynamic resistance in the two-phase flow of blood through small vertical tubes at low flow rates. Circ. Res. 68, 1–17 (1991). 1984854 10.1161/01.res.68.1.1

[b22] Fung Y. C. Stochastic flow in capillary blood vessels. Microvasc. Res. 5, 34–48 (1973). 4684755 10.1016/s0026-2862(73)80005-6

[b23] Park C. W., Shin S. H., Kim G. M., Jang J. H. & Gu Y. H. A hemodynamic study on a marginal cell depletion layer of blood flow inside a microchannel. Key Eng. Mater. 326, 863–866 (2006).

[b24] Fedosov D. A., Caswell B., Popel A. S. & Karniadakis G. E. Blood Flow and Cell‐Free Layer in Microvessels. Microcirculation. 17, 615–628 (2010). 21044216 10.1111/j.1549-8719.2010.00056.x PMC3529161

[b25] Chakraborty S. Dynamics of capillary flow of blood into a microfluidic channel. Lab Chip. 5, 421–430 (2005). 15791340 10.1039/b414566f

[b26] Yen R. T. & Fung Y. C. Effect of velocity distribution on red cell distribution in capillary blood vessels. Am. J. Physiol. Heart Circ. Physiol. 235, H251–H257 (1978). 10.1152/ajpheart.1978.235.2.H251 686194

[b27] Svanes K. & Zweifach B. W. Variations in small blood vessel hematocrits produced in hypothermic rats by micro-occlusion. Microvasc. Res. 1, 210–220 (1968).

[b28] Schmid-Schönbein G. W., Skalak R., Usami S. & Chien S. Cell distribution in capillary networks. Microvasc. Res. 19, 18–44 (1980). 7360046 10.1016/0026-2862(80)90082-5

[b29] Pries A. R., Secomb T. W. & Gaehtgens P. Biophysical aspects of blood flow in the microvasculature. Cardiovasc. Res. 32, 654–667 (1996). 8915184

[b30] Fenton B. M., Carr R. T. & Cokelet G. R. Nonuniform red cell distribution in 20 to 100 μm bifurcations. Microvasc. Res. 29, 103–126 (1985). 2580216 10.1016/0026-2862(85)90010-x

[b31] Faivre M., Abkarian M., Bickraj K. & Stone H. A. Geometrical focusing of cells in a microfluidic device: an approach to separate blood plasma. Biorheology. 43, 147–160 (2006). 16687784

[b32] Blattert C., Jurischka R., Schoth A., Kerth P. & Menz W. Separation of blood in microchannel bends. Proc. SPIE. 5345, 17–25 (2004). 10.1109/IEMBS.2004.1403754 17270814

[b33] Goldsmith H. L. & Marlow J. C. Flow behavior of erythrocytes. II. Particle motions in concentrated suspensions of ghost cells. J. Colloid Interface Sci. 71, 383–407 (1979).

[b34] Yang S., Ündar A. & Zahn J. D. A microfluidic device for continuous, real time blood plasma separation. Lab Chip. 6, 871–880 (2006). 16804591 10.1039/b516401j

[b35] Jäggi R. D., Sandoz R. & Effenhauser C. S. Microfluidic depletion of red blood cells from whole blood in high-aspect-ratio microchannels. Microfluid. Nanofluid. 3, 47–53 (2007).

[b36] Rodríguez-Villarreal A. I., Arundell M., Carmona M. & Samitier J. High flow rate microfluidic device for blood plasma separation using a range of temperatures. Lab Chip. 10, 211–219 (2010). 20066249 10.1039/b904531g

[b37] Kersaudy-Kerhoas M., Kavanagh D. M., Dhariwal R. S., Campbell C. J. & Desmulliez M. P. Validation of a blood plasma separation system by biomarker detection. Lab Chip. 10, 1587–1595 (2010). 20358050 10.1039/b926834k

[b38] Tripathi S., Prabhakar A., Kumar N., Singh S. G. & Agrawal A. Blood plasma separation in elevated dimension T-shaped microchannel. Biomed. Microdevices. 15, 415–425 (2013). 23355067 10.1007/s10544-013-9738-z

[b39] Prabhakar A., Kumar Y. V. B. V., Tripathi S. & Agrawal A. A novel, compact and efficient microchannel arrangement with multiple hydrodynamic effects for blood plasma separation. Microfluid. Nanofluid. 18, 995–1006 (2014).

[b40] Tripathi S., Kumar Y. V. B. V., Prabhakar A., Joshi S. S. & Agrawal A. Performance study of microfluidic devices for blood plasma separation–a designer’s perspective. J. Micromech. Microeng. 25, 084004 (2015).

[b41] Duryodhan V., Singh A., Singh S. G. & Agrawal A. A simple and novel way of maintaining constant temperature in microdevices. Sci. Rep. 6, 18230 (2016). 26795753 10.1038/srep18230 PMC4726283

[b42] Lee M. G. *et al.* Inertial blood plasma separation in a contraction–expansion array microchannel. Appl. Phys. Lett. 98, 253702 (2011). 10.1016/j.chroma.2010.11.081 21176909

[b43] Nivedita N. & Papautsky I. Continuous separation of blood cells in spiral microfluidic devices. Biomicrofluidics. 7, 054101(2013). 10.1063/1.4819275 PMC3779264 24404064

[b44] Di Carlo D., Edd J. F., Irimia D., Tompkins R. G. & Toner M. Equilibrium separation and filtration of particles using differential inertial focusing. Anal. Chem. 80, 2204–2211 (2008). 18275222 10.1021/ac702283m

[b45] Martel J. M. & Toner M. Inertial focusing in microfluidics. Annu. Rev. Biomed. Eng. 16, 371 (2014). 24905880 10.1146/annurev-bioeng-121813-120704 PMC4467210

[b46] Martel J. M. & Toner M. Particle focusing in curved microfluidic channels. Sci. Rep. 3, 3340 (2013).

[b47] Lim E. J., Ober T. J., Edd J. F., McKinley G. H. & Toner M. Visualization of microscale particle focusing in diluted and whole blood using particle trajectory analysis. Lab Chip. 12, 2199–2210 (2012). 22382737 10.1039/c2lc21100a PMC4211080

[b48] Martel Joseph M. *et al.* Continuous Flow Microfluidic Bioparticle Concentrator. Sci. Rep. 5, 11300 (2015). 26061253 10.1038/srep11300 PMC4462155

[b49] Wang T., Rongin U. & Xing Z. A micro-scale simulation of red blood cell passage through symmetric and asymmetric bifurcated vessels. Sci. Rep. 6, 20262 (2016). 26830454 10.1038/srep20262 PMC4735796

[b50] Paul R. *et al.* Shear stress related blood damage in laminar couette flow. Artif. Organs. 27, 517–529 (2003). 12780506 10.1046/j.1525-1594.2003.07103.x

[b51] Chang W., Tzebotich D., Lee L. P. & Liepmann D. Blood flow in simple microchannels. *Microtechnologies in Medicine and Biology, 1st Annual International, Conference On.* 2000, IEEE, 311–315 (2000).

[b52] Zaia D. A. M., Zaia C. T. B. V. & Lichtig J. Determination of total protein by spectrophotometry: advantages and disadvantages of proposed methods. Quím. Nova. 21, 787–793 (1998).

[b53] Gornall A. G., Bardawill C. J. & David M. M. Determination of serum proteins by means of the biureto reaction. J. Biol. Chem. 177, 751–766 (1949). 18110453

[b54] Keller R. P. & Neville M. C. Determination of total protein in human milk: comparison of methods. Clin. Chem. 32, 120–123 (1986). 3940691

[b55] Cengiz E. & Tamborlane W. V. A tale of two compartments: interstitial versus blood glucose monitoring. Diabetes Technol. Ther. 11, S11–S16 (2009). 19469670 10.1089/dia.2009.0002 PMC2903977

[b56] Kemna E. W. *et al.* High-yield cell ordering and deterministic cell-in-droplet encapsulation using Dean flow in a curved microchannel. Lab Chip. 12, 2881–2887 (2012). 22688131 10.1039/c2lc00013j

[b57] Bhagat A. A. S., Kuntaegowdanahalli S. S. & Papautsky I. Continuous particle separation in spiral microchannels using dean flows and differential migration. Lab Chip. 8, 1906–1914 (2008). 18941692 10.1039/b807107a

[b58] Tripathi S., Kumar A., Kumar Y. B. V. & Agrawal A. Three-dimensional hydrodynamic flow focusing of dye, particles and cells in a microfluidic device by employing two bends of opposite curvature. Microfluid. Nanofluid. 20, 1–14 (2016).

[b59] Di Carlo D., Irimia D., Tompkins R. G. & Toner M. Continuous inertial focusing, ordering, and separation of particles in microchannels. Proc. Natl. Acad. Sci. USA 104, 18892–18897 (2007). 18025477 10.1073/pnas.0704958104 PMC2141878

[b60] Cengel Y. A. Fluid mechanics. 2nd edn. Tata McGraw-Hill Education (2010).

[b61] Soedjak H. S. Colorimetric micromethod for protein determination with erythrosin B. Anal. Biochem. 220, 142–148 (1994). 7978237 10.1006/abio.1994.1310

[b62] Cole L. A. “Background” Human Chorionic Gonadotropin in Healthy, Nonpregnant Women. Clin. Chem. 51, 1765–1766 (2005). 16189375 10.1373/clinchem.2005.056507

[b63] Rennke H. G. & Denker B. M. Renal pathophysiology: the essentials. 2nd edn. Lippincott Williams & Wilkins (2007).

[b64] Mach A. J. & Di Carlo D. Continuous scalable blood filtration device using inertial microfluidics. Biotechnol. Bioeng. 107, 302–311 (2010). 20589838 10.1002/bit.22833

[b65] Sollier E., Cubizolles M., Fouillet Y. & Achard J. L. Fast and continuous plasma extraction from whole human blood based on expanding cell-free layer devices. Biomed. Microdevices. 12, 485–497 (2010). 20204703 10.1007/s10544-010-9405-6

