# Supplementary material for: Microdevice for plasma separation from whole human blood using bio-physical and geometrical effects
Source: Sci Rep. 2016 Jun 9;6:26749. doi: 10.1038/srep26749 (PMC4899686; doi:10.1038/srep26749)
Supplement: Supplementary Information [file srep26749-s1.pdf]

Title: Microdevice for plasma separation from whole human blood using bio-physical and geometrical effects

SREP-15-34707 B

By: Siddhartha Tripathi, Y.V. Bala Varun Kumar, Amit Agrawal, Amit Prabhakar, and Suhas S Joshi

Video Legends

Video 1:

Blood plasma separation with whole blood at 0.5 ml/min

Video 2:

Demonstration of clog free operation

Video 3:

Blood plasma separation at high hematocrit-Hct 62% at 0.5 ml/min
